# Correction: The AMPA receptor-associated protein Shisa7 regulates hippocampal synaptic function and contextual memory

**DOI:** 10.7554/eLife.36711

**Published:** 2018-05-04

**Authors:** Leanne J M Schmitz, Remco V Klaassen, Marta Ruiperez-Alonso, Azra Elia Zamri, Jasper Stroeder, Priyanka Rao-Ruiz, Johannes C Lodder, Rolinka J van der Loo, Huib D Mansvelder, August B Smit, Sabine Spijker

Schmitz LJM, Klaassen RV, Ruiperez-Alonso M, Zamri AE, Stroeder J, Rao-Ruiz P, Lodder JC, van der Loo RJ, Mansvelder HD, Smit AB, Spijker S. 2017. The AMPA receptor-associated protein Shisa7 regulates hippocampal synaptic function and contextual memory. *eLife* **6**:e24192. doi: 10.7554/eLife.24192.

Published 4, December 2017

In the original article, the blots shown in Figure 1d had been previously published (Figure 2d in Klaassen et al., 2016). We realize that this should have been clearly indicated in the figure legend and we apologize to the readers for this. For context, the subcellular distribution analysis of Shisa7 (Figure 1d) had been performed in parallel with that of the previously published analysis for Shisa6 (Klaassen et al., 2016) to allow comparisons between these two family members, and we re-used the blots of the "marker proteins" (GluA2, PSD-95, GluN2A and Syp) to show enrichment. We now replace these blots with original data for GluA2, PSD-95, GluN2B and Syp, which were acquired at the time of the original experiment, using the same biochemical enrichment fractions. The conclusion regarding the subcellular distribution profile of Shisa7 remains the same. The corrected version of Figure 1d with replacement of the “marker blots” to show subcellular enrichment is shown below:

**Figure 1.**
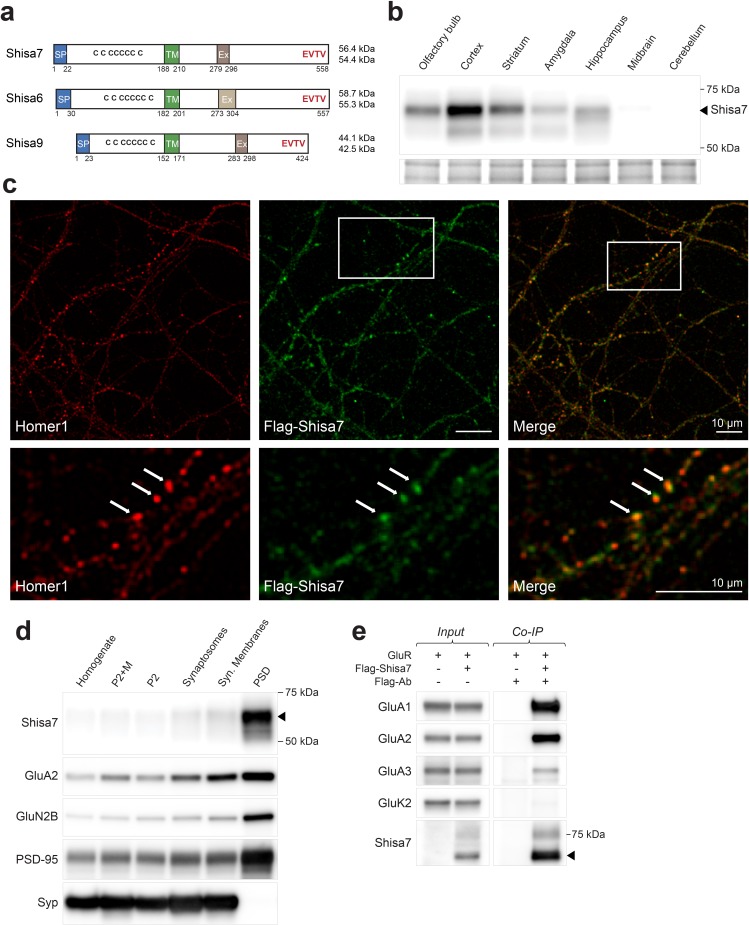
Shisa7 is a type-I transmembrane protein interacting with AMPA-type receptors. d) Biochemical fractionation (homogenate (H), crude synaptic membranes (P2; with and without microsomes (M)), synaptosomes (SS), synaptic membranes (SM) and postsynaptic density fraction (PSD; Triton X-100 insoluble fraction) of mature mouse hippocampus reveals an enrichment of Shisa7 in the PSD together with GluA2, GluN2B, PSD-95, and this pattern is distinct from the presynaptic marker Synaptophysin (Syp).

**Figure 1—figure supplement 3.**
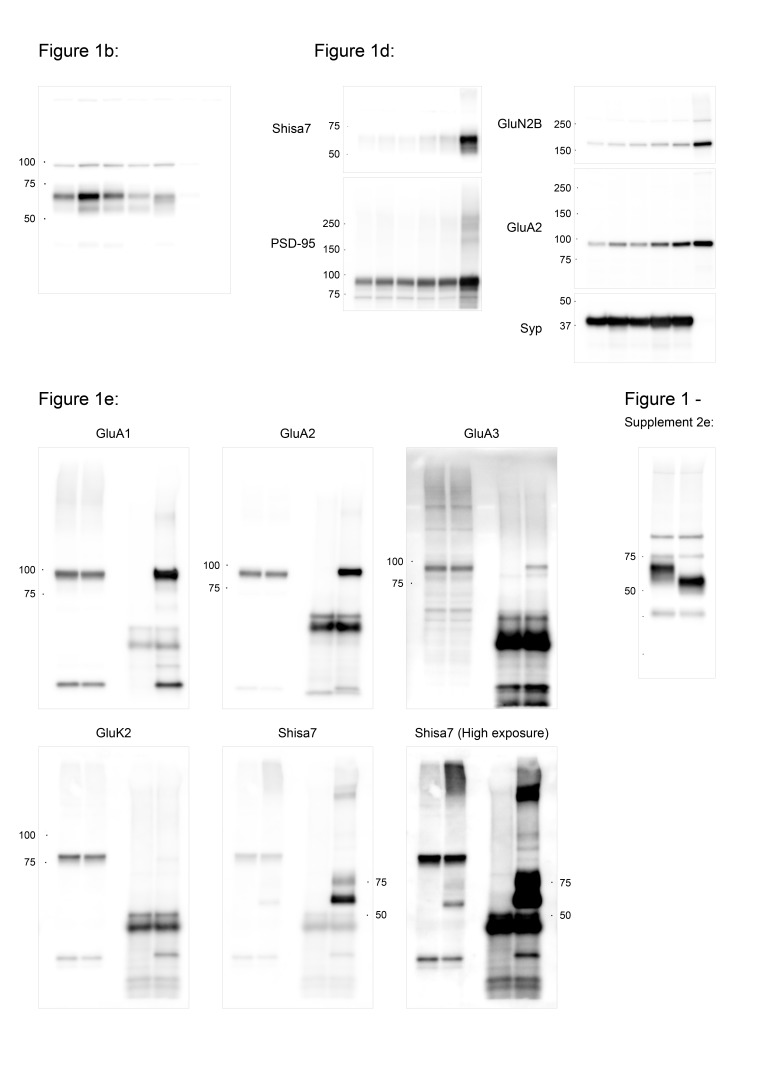


The corrected legend for Figure 1d follows:

**Figure 1. Shisa7 is a type-I transmembrane protein interacting with AMPA-type receptors**. d) Biochemical fractionation (homogenate (H), crude synaptic membranes (P2; with and without microsomes (M)), synaptosomes (SS), synaptic membranes (SM) and postsynaptic density fraction (PSD; Triton X-100 insoluble fraction) of mature mouse hippocampus reveals an enrichment of Shisa7 in the PSD together with GluA2, GluN2B, PSD-95, and this pattern is distinct from the presynaptic marker Synaptophysin (Syp).

Figure 1—figure supplement 3 has been updated accordingly with the full blots of the replacement “marker blots” for Figure 1d:

We also correct mistakes in Figure 4a and Figure 4—figure supplement 1a. In the original figures, the bar graphs for Shisa9 were different between the two figures whereas they should have been identical. After checking the original data we found that the height of the bars represented in Figure 4a are incorrect. In addition, the example blots of GluA2 erroneously duplicated the blot of GluA1. To correct these errors we have remade both figures from the raw data.

The revised versions of Figure 4a and Figure 4—figure supplement 1a with the correct placement of the GluA2 blots are shown below: 

**Figure 4.**
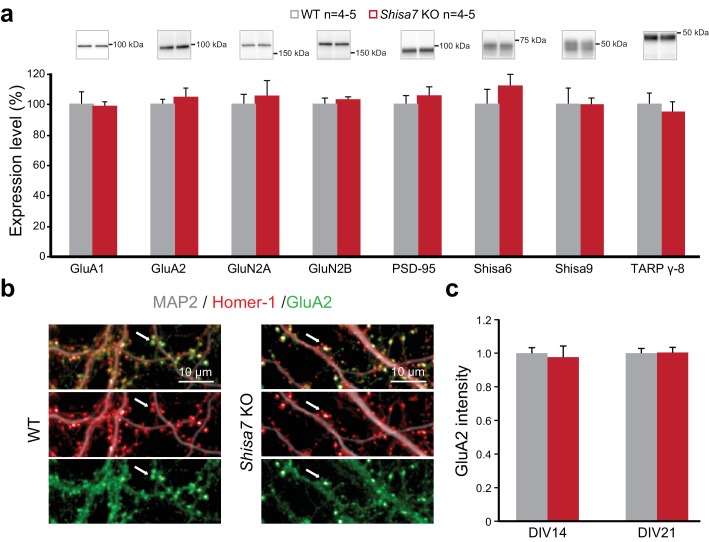


**Figure 4—figure supplement 1.**
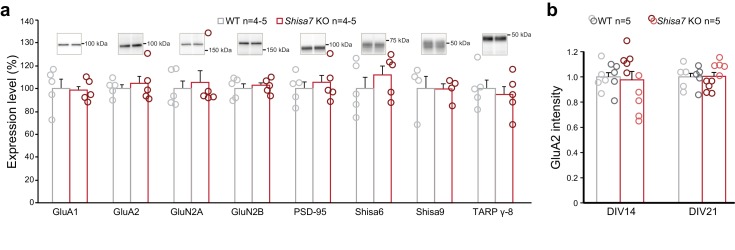


Finally, we correct a mistake in the labelling of Figure 6d and Figure 6—figure supplement 2b. The main Figure (d) and the figure supplement (b) indicated ‘GluA2’. However, this should have been labelled ‘GluA1’, as was correctly mentioned in the figure legends and the Materials and Methods.

The article has been corrected accordingly.
