# Supplementary material for: The AMPA receptor-associated protein Shisa7 regulates hippocampal synaptic function and contextual memory
Source: eLife. 2017 Dec 4;6:e24192. doi: 10.7554/eLife.24192 (PMC5737659; doi:10.7554/eLife.24192)
Supplement: Supplementary file 1. — In vitro data on TARP γ−8 are from Milstein et al. (2007). The Shisa9 and TARP γ−8 ex/in vivo data originate from Khodosevich et al. (2014), in which experiments were performed using WT, KO and overexpression conditions in the DG, which is regionally more appropriate for Shisa9 than overexpression in CA1 (von Engelhardt et al., 2010), and from the CA1 for TARP γ−8 KO (Rouach et al., 2005). The Shisa6 ex/in vivo data stem from WT vs. KO comparisons in CA1 pyramidal cells activated by (electrically-evoked) CA3 input (Schaffer collaterals (Klaassen et al., 2016)), similar as used for Shisa7 (bold). NA: Not applicable, not measured. [file elife-24192-supp1.docx]

| **Parameter** | **Shisa9** | **Shisa6** | | **Shisa7** | **TARP γ-8** | | **Method used** |
| --- | --- | --- | --- | --- | --- | --- | --- |
| ***In vitro*** | | | | | | |  |
| Amplitude | Enhanced | Not affected | Not affected | | NA | | 9: Oocytes (Engelhardt et al., 2010)  6&**7**: HEK293 (Klaassen et al., 2016) |
| Rise time | Not affected | Not affected | Not affected | |  | | 9: Oocytes (Engelhardt et al., 2010)  6&**7**: HEK293 (Klaassen et al., 2016)  g8: HEK293 outside-out patch (Milstein et al., 2007) |
| Deactivation | Slower (increased tau) | Slower (increased tau) | Not affected | | Slower (increased tau) | | 9: Oocytes (Engelhardt et al., 2010)  6&**7**: HEK293 (Klaassen et al., 2016)  g8: HEK293 outside-out patch(Milstein et al., 2007) |
| Desensitiz. | Faster (decreased tau) | Slower (increased tau) | Faster (decreased tau) | | Slower (increased tau) | | 9: HEK293 (Khodosevich et al., 2014)  6&**7**: HEK293 (Klaassen et al., 2016)  g8: HEK293 outside-out patch(Milstein et al., 2007) ; HEK293 (Khodosevich et al., 2014) |
| Steady-state conduct. | Decreased | Enhanced | Decreased | | Enhanced | | 9: HEK293(Khodosevich et al., 2014)  6&**7**: HEK293 (Klaassen et al., 2016)  g8: HEK293 outside-out patch(Milstein et al., 2007) ; HEK293 (Khodosevich et al., 2014) |
| Rec. from desensitiz. | Slower (increased tau) | Slower (increased tau) | Slower (increased tau) | | NA | | 9: HEK293 (Khodosevich et al., 2014)  6&**7**: HEK293 (Klaassen et al., 2016) |
|  | | | | | | | |
| **Parameter** | **Shisa9** | **Shisa6** | **Shisa7** | | **TARP γ-8** | |  |
|  | **DG** | **CA1** | **CA1** | | **DG** | **CA1** | **Method used** |
| ***Ex/in vivo*** | | | | | | |  |
| Amplitude; surface expression | Enhanced | Not affected | Not affected | | Enhanced | Enhanced (extra-synaptic) | 9: Acute slice (DG; mEPSC, EPSC, Input-output curves), Outside-out patch (DG), GluA1 cluster analysis(Khodosevich et al., 2014)  6&**7**: Acute slice (CA1; mEPSC & evoked EPSC), Immunoblotting (synaptic membranes)(Klaassen et al., 2016)  **7**: Primary culture  g8: Acute slice (DG; mEPSC, EPSC, Input-output curves), Outside-out patch (DG), GluA1 cluster analysis(Khodosevich et al., 2014); Slice culture (CA1, overexpression), acute slice (CA1), immunoblotting (hippocampus, KO)(Rouach et al., 2005) |
| Rise time | Not affected | Enhanced | Enhanced | | Enhanced |  | 9: outside-out patch (DG)(Khodosevich et al., 2014)  6&**7**: Acute slice (CA1, mEPSC)(Klaassen et al., 2016)  g-8: outside-out patch in KO (DG)(Khodosevich et al., 2014) |
| Decay time | Slower (increased tau) | Slower (increased tau) | Slower (increased tau) | | Slower (increased tau) |  | 9: outside-out patch (DG)(Khodosevich et al., 2014)  6&**7**: Acute slice (CA1, mEPSC)(Klaassen et al., 2016)  6: Acute slice (CA1, dendritic glutamate uncaging)(Klaassen et al., 2016)  **7**: Acute slice (CA1, evoked EPSCs)  g-8: outside-out patch in KO (DG)(Khodosevich et al., 2014) |
| Frequency | Enhanced | Not affected | Not affected | | Enhanced |  | 9: Acute slice (DG, mEPSCs)(Khodosevich et al., 2014)  6&**7**: Acute slice (CA1, mEPSC)(Klaassen et al., 2016)  g-8: Acute slice (DG, mEPSCs)(Khodosevich et al., 2014) |
| Desensitiz. | Faster (decreased tau) | NA | NA | | Slower (increased tau) |  | 9: outside-out patch (DG)(Khodosevich et al., 2014)  g-8: outside-out patch in KO (DG)(Khodosevich et al., 2014) |
| Rec. from desensitiz. | Slower (increased tau) | NA | NA | | Faster (decreased tau) |  | 9: outside-out patch (DG)(Khodosevich et al., 2014)  g-8: outside-out patch in KO (DG)(Khodosevich et al., 2014) |
| Steady-state conduct. | Decreased | NA | NA | | Not affected |  | 9: outside-out patch (DG)(Khodosevich et al., 2014)  g-8: outside-out patch in KO (DG)(Khodosevich et al., 2014) |
| Paired pulse ratio | Decreased^1,2^ | Not affected | Not affected | | Enhanced | Enhanced | 9: Acute slice (DG, evoked EPSCs)(Engelhardt et al., 2010; Khodosevich et al., 2014) in KO  6&**7**: Acute slice (CA1, evoked EPSCs)(Klaassen et al., 2016)  g-8: Acute slice (DG, evoked EPSCs)(Khodosevich et al., 2014); Acute slice (CA1)(Rouach et al., 2005) |
| Short-term plasticity | Decreased | Enhanced | Not affected | | Enhanced |  | 9: Acute slice (DG, evoked EPSCs)(Khodosevich et al., 2014) in KO  6&**7**: Acute slice (CA1, evoked EPSCs)(Klaassen et al., 2016)(present paper)  g-8: Acute slice (DG, evoked EPSCs)^2^ |
| Long-term plasticity | Unaffected | NA | Enhanced | | Enhanced | Enhanced | 9: Acute slice (DG, low frequency pairing)(Khodosevich et al., 2014)  **7**: Acute slice (CA1; theta burst)  g-8: Acute slice (DG, low frequency pairing)(Khodosevich et al., 2014); Acute slice (CA1; tetanic stimulation)(Rouach et al., 2005) |
