# Supplementary material for: The AMPA receptor-associated protein Shisa7 regulates hippocampal synaptic function and contextual memory
Source: eLife. 2017 Dec 4;6:e24192. doi: 10.7554/eLife.24192 (PMC5737659; doi:10.7554/eLife.24192)
Supplement: Transparent reporting form [file elife-24192-transrepform.docx]

***eLife’s* transparent reporting form**

We encourage authors to provide detailed information *within their submission* to facilitate the interpretation and replication of experiments. If you have any questions, please contact us: [editorial@elifesciences.org](mailto:editorial@elifesciences.org).

**Sample-size estimation**

- You should state whether an appropriate sample size was computed when the study was being designed
- You should state the statistical method of sample size computation and any required assumptions
- If no explicit power analysis was used, you should describe how you decided what sample (replicate) size (number) to use

Please outline where this information can be found within the submission (e.g., page numbers or figure legends), or explain why this information doesn’t apply to your submission:

In all legends the n-number (cells and/or animals) used is mentioned; for quantitative analysis of PCR (Fig. 1-S1), immunoblotting (Fig. 1-S2; Fig. 1b, Fig. 4a; Fig. 4-S1), primary culture (Fig. 1c; Fig. 4b,c; Fig. 4-S1; Fig. 6d,e), electrophysiology (HEK cells [Fig. 2; Fig. 2-S1], slices [Fig. 3; Fig. 3-S1; Fig. 5; Fig. 5-S1; Fig. 6a-c; Fig. 6-S1, -S2]) and behavioral measurements (Fig. 7; Fig. 7-S1, -S2) the sample size was based on previous experiments.

**Replicates**

- You should report how often each experiment was performed
- You should include a definition of biological versus technical replication
- The data obtained should be provided and sufficient information should be provided to indicate the number of independent biological and/or technical replicates
- If you encountered any outliers, you should describe how these were handled
- Criteria for exclusion/inclusion of data should be clearly stated
- High-throughput sequence data should be uploaded before submission, with a private link for reviewers provided (these are available from both GEO and ArrayExpress)

Please outline where this information can be found within the submission (e.g., page numbers or figure legends), or explain why this information doesn’t apply to your submission:

In the legends the n-number (cell, animals; technical/biological replicates) used is mentioned; for quantitative analysis of qPCR and normal PCR (Fig. 1-S1), for primary cultures (Fig. 4-S1; Fig. 6; Fig. 6-S2).

Criteria for omitting data from HEK cells (electrophysiological recordings) and for omitting wells for culture experiments are mentioned in the Materials and methods. In addition, the effect of omitting an outlier for behavioral analysis (Fig. 7-S2) is mentioned.

**Statistical reporting**

- Statistical analysis methods should be described and justified
- Raw data should be presented in figures whenever informative to do so (typically when N per group is less than 10)
- For each experiment, you should identify the statistical tests used, exact values of N, definitions of center, methods of multiple test correction, and dispersion and precision measures (e.g., mean, median, SD, SEM, confidence intervals; and, for the major substantive results, a measure of effect size (e.g., Pearson's r, Cohen's d)
- Report exact p-values wherever possible alongside the summary statistics and 95% confidence intervals. These should be reported for all key questions and not only when the p-value is less than 0.05.

All statistical tests are indicated upon mentioning the exact *P*-values in the result section for any given analysis. The justification for statistical testing is indicated in the MM section (last section).

Data is indicated in Table 1 for Shisa7 interaction partners for Glutamate receptor subunits, established non-PDZ AMPAR interacting proteins (Schwenk et al., 2012) and known PDZ-containing interactors for the n=3 experiment (KO and WT each). The Table 1-S1 yields full data of this experiment, and Table 1-S2 provides the statistical analysis (see below).

Please outline where this information can be found within the submission (e.g., page numbers or figure legends), or explain why this information doesn’t apply to your submission:

(For large datasets, or papers with a very large number of statistical tests, you may upload a single table file with tests, Ns, etc., with reference to page numbers in the manuscript.)

**Additional data files (“source data”)**

- We encourage you to upload relevant additional data files, such as numerical data that are represented as a graph in a figure, or as a summary table
- Where provided, these should be in the most useful format, and they can be uploaded as “Source data” files linked to a main figure or table
- Include model definition files including the full list of parameters used
- Include code used for data analysis (e.g., R, MatLab)
- Avoid stating that data files are “available upon request”

Please indicate the figures or tables for which source data files have been provided:

Table 1-S1, -S2.
